# Bloodless Amputations

**Published:** 1845-07

**Authors:** 


### BLOODLESS AMPUTATIONS.

Our friend, Dr. Mosby, proposes to save the subjects of amputations from loss of blood, occasionally a very disastrous circumstance in exhausted individuals, by the following method, which he lately communicated to us, and requests us to submit to the profession. First, he would apply a roller bandage to the limb, so as to force the blood as much as possible out of it, and then, by means of a tournequet, cut off the ingress of blood by the arteries, slackening the instrument after the amputation sufficiently to find the vessels that might bleed.

Our worthy friend has made some ingenious suggestions to us in relation to the treatment of hemorrhoidal tumors, which we will communicate in a future number of the Journal. Y.

